# Supplementary material for: Engineering of a bona fide light-operated calcium channel
Source: Nat Commun. 2021 Jan 11;12:164. doi: 10.1038/s41467-020-20425-4 (PMC7801460; doi:10.1038/s41467-020-20425-4)
Supplement: Supplementary file 1 — Supplementary Information [file 41467_2020_20425_MOESM1_ESM.pdf]

Supporting information for

Engineering of a *bona fide* light-operated calcium channel

Lian He, Liuqing Wang, Hongxiang Zeng, Peng Tan, Guolin Ma, Sisi Zheng, Yaxin Li, Lin Sun, Fei Dou, Stefan Siwko, Yun Huang, Youjun Wang, Yubin Zhou

Contents:

Supplementary Figures 1-6
Supplementary Movies 1-4
Supplementary Tables 1-2
Supplementary References

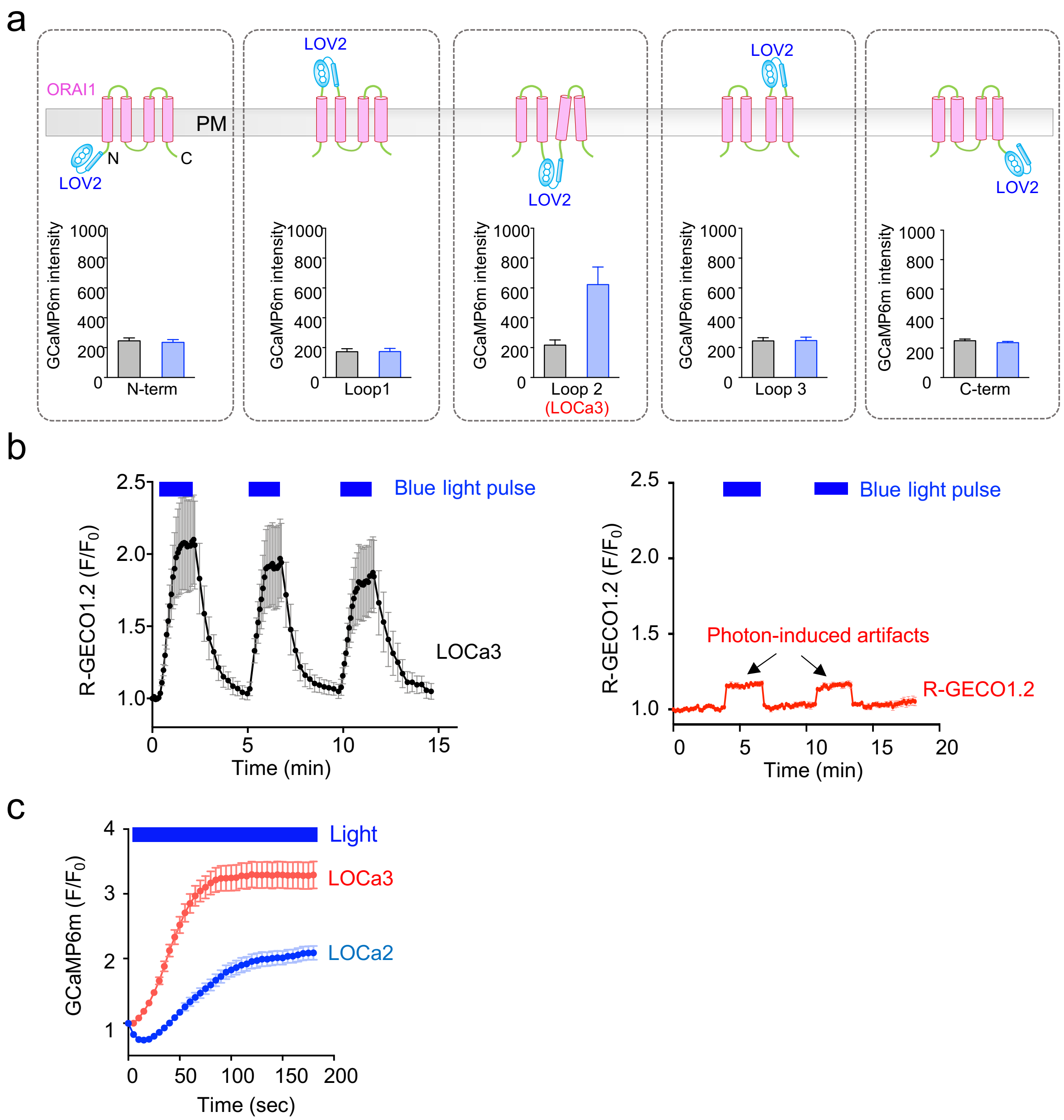

Supplementary Figure 1 | Installing optogenetic modules into ORAI1 to generate light-operated Ca^{2+} channels (LOCa).

Data were shown as mean ± s.e.m.. Blue light was delivered at 470 nm with a power density of 40 µW/mm^2.

(a) Strategies employed to design photoswitchable ORAI1 Ca^{2+} channels. GCaMP6m signals before and after blue light illumination were plotted as bar graphs below the cartoons. n = 16-40 cells.

(b) Reversible control of Ca^{2+} signals in HEK293 cells. LOCa3-expressing cells were exposed to three repeated light-dark cycles, with the intracellular Ca^{2+} changes monitored by R-GECO1.2 (left panel; n = 10 cells). The artifacts for R-GECO1.2 caused by blue light were also shown as control (right panel; n = 8 cells).

(c) Representative time courses of light-induced Ca^{2+} entry in HEK293 cells expressing LOCa2 or LOCa3. GCaMP6m was co-expressed to report cytosolic Ca^{2+} changes upon light stimulation. The GCaMP6m signal statured after about 90 sec of blue light illumination (LOCa3); while LOCa2 took about 130 sec. The activation half-lives were determined to be: 53.6 ± 2.7 sec (second phase; LOCa2) and 34.2 ± 1.5 sec (LOCa3). n = 52 cells from three independent assays.

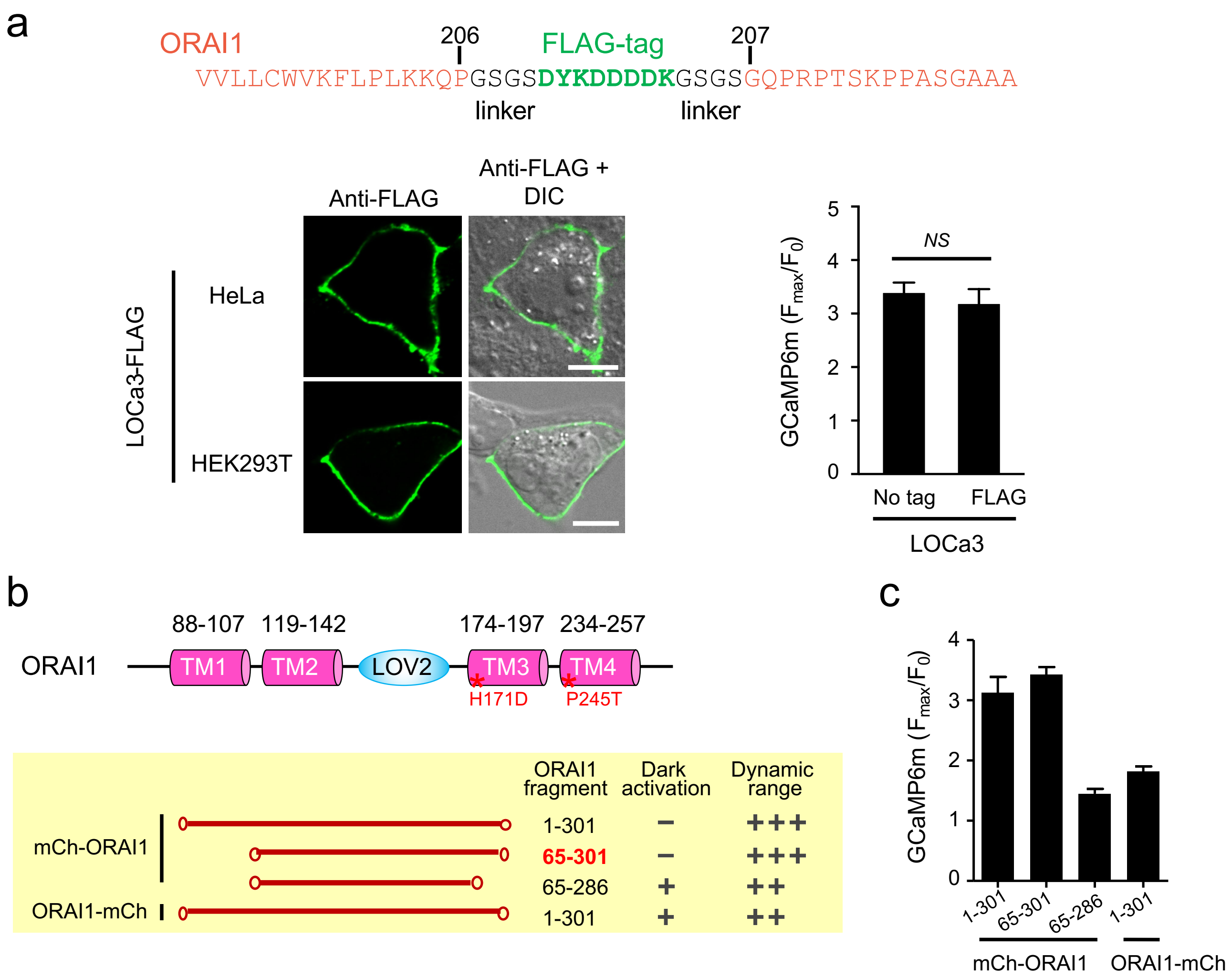

Supplementary Figure 2 | Characterization of tag-fused and truncated LOCa3.

Data were shown as mean ± s.e.m.. Blue light was delivered at 470 nm with a power density of 40 μW/mm^2.

(a) Confocal images of intact HeLa or HEK293T cells expressing LOCa3 with a FLAG tag inserted in the second extracellular loop between residues 206 and 207 with a GSGS linker on either side (left panel). Cells were left unpermeabilized and stained with an anti-FLAG antibody. Scale bar, 10 μm. The primary sequence of the LOCa3-FLAG junction region was shown above the confocal images. Right panel, FLAG insertion into LOCa3 did not significantly affect light induced Ca^{2+} influx in HeLa cells. n = 30 cells.

(b) Summary of the light-dependent changes in cytosolic Ca^{2+} for the indicated LOCa3 truncation or deletion variants.

(c) Quantification of light-induced changes in GCaMP6m signals for HEK293 cells expressing the indicated constructs. n = 36-52 cells.

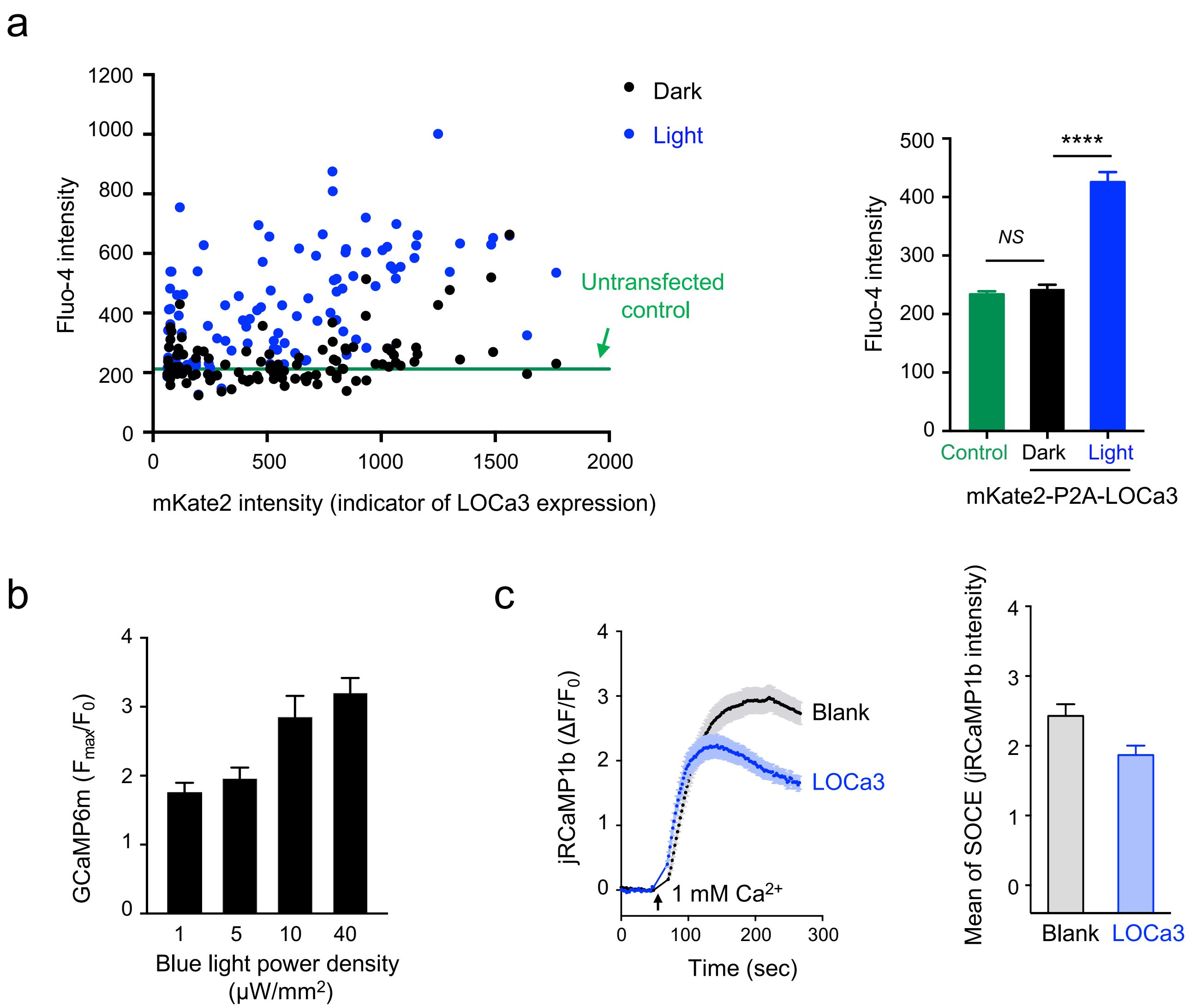

Supplementary Figure 3 | Characterization of photoactivatable Ca^{2+} entry mediated by LOCa3.

Data were shown as mean ± s.e.m..

(a) Quantification of light induced intracellular Ca^{2+} changes in HeLa cells expressing mKate2-P2A-LOCa3 at varying degrees (indicated by mKate2 fluorescence intensities). The averaged Fluo-4 background signal (mKate2 negative cells) was indicated by the green line (negative control). The Fluo-4 signals for individual mKate2 positive cells before (dark) and after light stimulation (blue) were plotted in the left panel. In the dark state, the majority of LOCa3-expressing cells showed no or little background activation. Right panel, quantification of light-induced changes in Fluo-4 intensity. No significant difference was noted between LOCa3-transfected or non-transfected cells (n = 77-105 cells). ****P < 0.001 when compared to the dark group, P = 0.4433 for Control and Dark group, two tailed unpaired Student's *t*-test.

(b) Light-tunable Ca^{2+} entry in LOCa3-expressing HeLa cells. Maximal fold-changes of GCaMP6m signals were plotted against varying light power densities at 470 nm (n = 25-31 cells).

(c) Evaluation of LOCa3 expression on endogenous SOCE responses in HEK293 cells. To deplete ER Ca^{2+} store, cells were kept in nominally Ca^{2+} free imaging solution containing 1 μM thapsigargin (TG) for 10 min. TG was present throughout the recordings. Compared to the control, TG-induced SOCE response in LOCa3-expressing cells was slightly reduced (n = 12 cells).

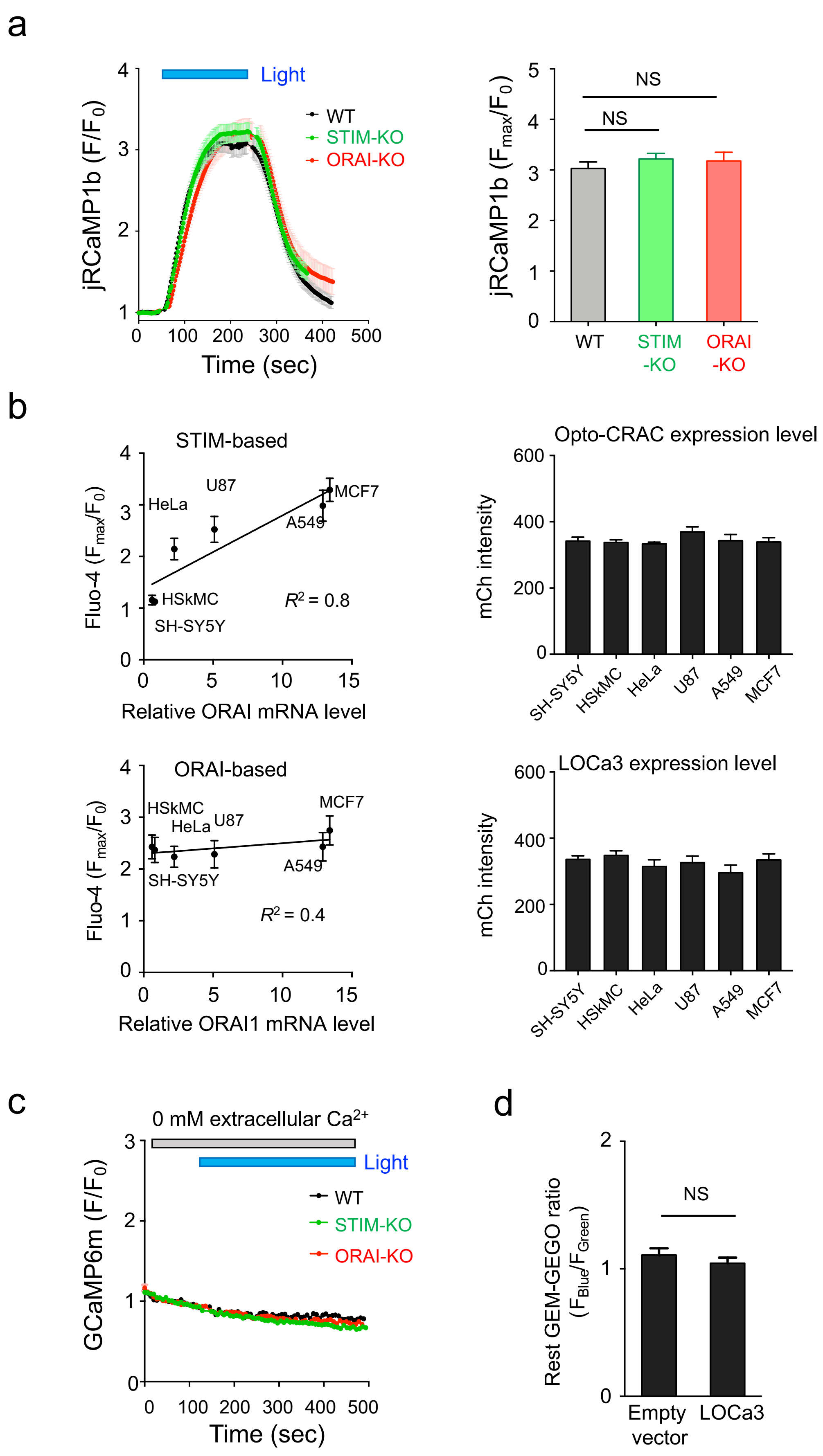
a
Light
WT
STIM-KO
ORAI-KO
jRCaMP1b (F/F0)
Time (sec)
NS
NS
jRCaMP1b (Fmax/F0)
WT
STIM -KO
ORAI -KO
b
STIM-based
HeLa
U87
MCF7
A549
HSkMC
SH-SY5Y
R2 = 0.8
Fluo-4 (Fmax/F0)
Relative ORAI mRNA level
Opto-CRAC expression level
mCh intensity
SH-SY5Y
HSkMC
HeLa
U87
A549
MCF7
ORAI-based
HSkMC
HeLa
U87
MCF7
A549
SH-SY5Y
R2 = 0.4
Relative ORAI1 mRNA level
LOCa3 expression level
c
0 mM extracellular Ca2+
Light
WT
STIM-KO
ORAI-KO
GCaMP6m (F/F0)
Time (sec)
d
NS
Rest GEM-GEGO ratio (FBlue/FGreen)
Empty vector
LOCa3

Supplementary Figure 4 | Activation of LOCa3 is independent of endogenous STIM and ORAI expression levels.

Data were shown as mean ± s.e.m.. Blue light was delivered at 470 nm with a power density of 40 μW/mm^2.

(a) Reversible control of Ca^{2+} signals in WT, STIM-KO (STIM1-STIM2 double KO) or ORAI-KO (ORAI1, ORAI2 and ORAI3 triple KO) in LOCa3-expressing HEK293T cells. The bar graph showed the peak values of jRCaMP1b fluorescence (n = 14-20 cells).

(b) STIM1-based Opto-CRAC, but not LOCa3, exhibited an ORAI1 expression level-dependent response in Ca^{2+} signals upon photostimulation. Left panel, blue light-induced maximal changes in the Fluo-4 Ca^{2+} indicator signals mediated by Opto-CRAC (upper) or ORAI1-based LOCa3 (lower) in the indicated cell types with different endogenous ORAI1 levels. Ca^{2+} changes reported by Fluo-4 were plotted against normalized ORAI1 mRNA expression levels; Right panel, cell populations with similar expression levels were selected for the analysis in order to avoid potential concentration-dependent artifacts. n = 10-19 cells.

(c) Typical traces showing that blue light illumination induced no appreciable increases in GCaMP6m signals when cells were bathed in an extracellular solution containing 0 mM Ca^{2+}. n= 59-71 cells

(d) The resting cytosolic Ca^{2+} levels of LOCa3 in HEK293T cells. A highly sensitive ratio-metric Ca^{2+} indicator, GEM-GECO, together with an empty vector (as control) or LOCa3, were transiently expressed in HEK293T cells, and the resting GEM-GECO ratio was calculated. No significant difference was noted between the two groups. n= 22-38 cells.

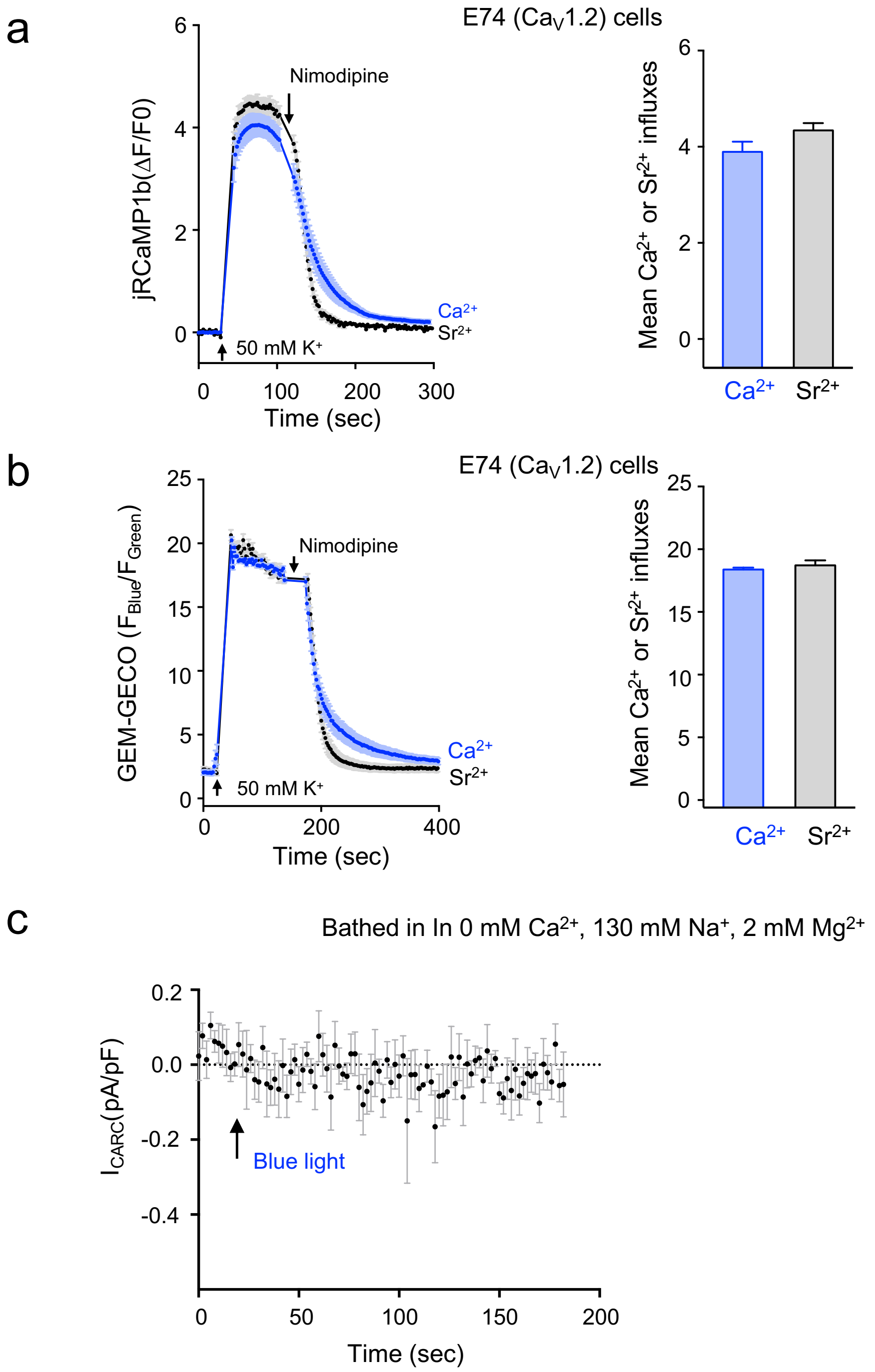

Supplementary Figure 5 | Characterizations of Ca^{2+} selectivity for LOCa3 and $Ca_V1.2$ channels.

(a-b) In HEK-E74 $Ca_V1.2$-expressing cells transiently expressing jRCaMP1b (a) or GEM-GECO (b), high K^+-induced Ca^{2+} (blue) or Sr^{2+} (black) influxes through $Ca_V1.2$ channels were examined. Left, typical traces; right, statistics. 2 μM nimodipine was used to block the activity of $Ca_V1.2$ channels. No significant difference between mean Ca^{2+} (blue) or Sr^{2+} (black) entry was found (*P* = 0.1011 for (a), *P* = 0.3525 for (b); two-tailed Student's *t*-test; n=15 cells from three independent experiments). Data were shown as mean ± s.e.m..

(c) When bathed in nominally Ca^{2+} free solution, HEK293 OK (ORAI-knockout) cells expressing LOCa3 showed no detectable light-induced Na^+ current. *Left*, the mean time-course of whole-cell current recording; *Right,* the mean current-voltage relationship at the end of electrophysiological recording shown on the left (n = 5 cells). Data were shown as mean ± s.e.m..

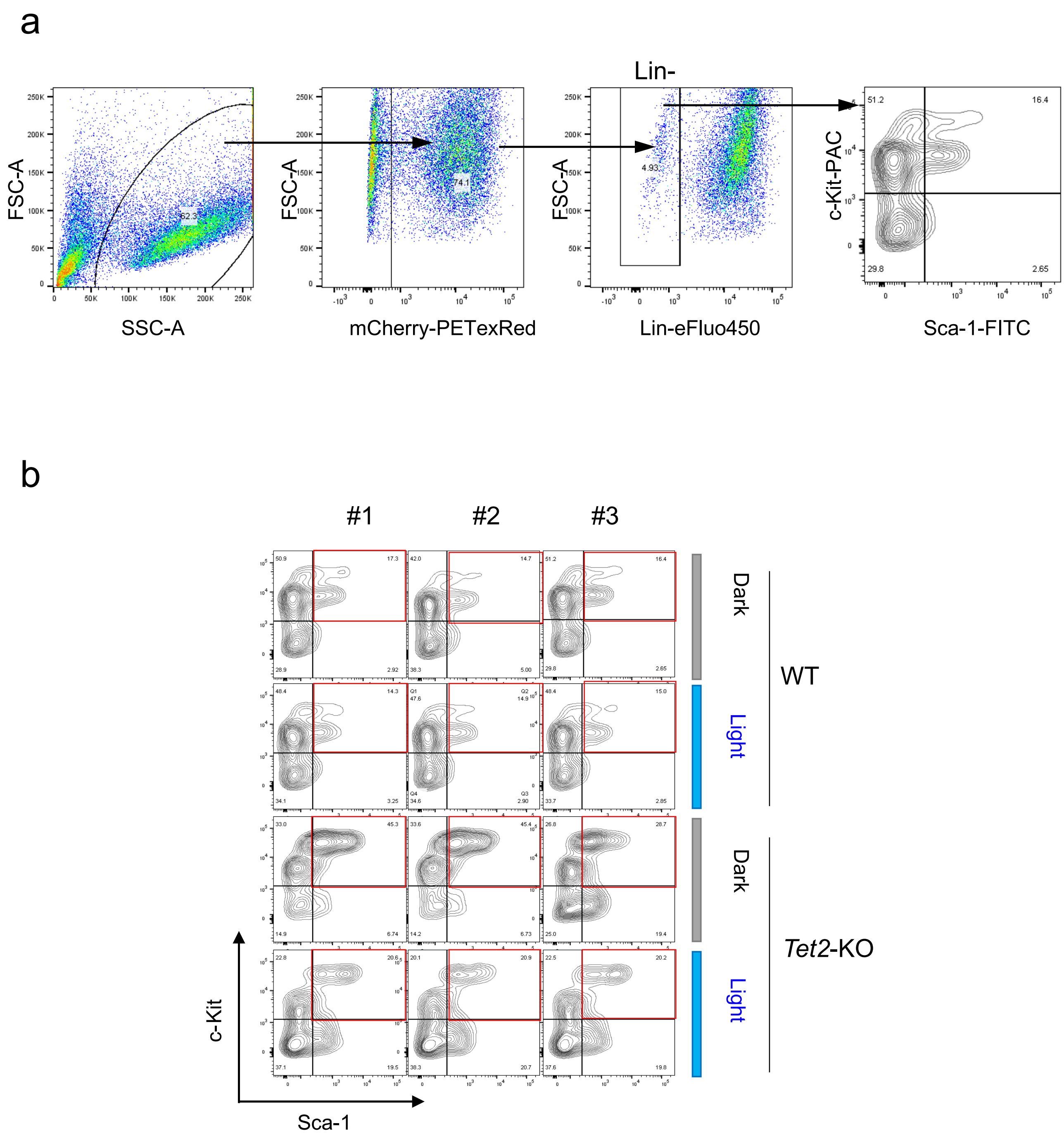

Supplementary Figure 6 | Flow cytometry analysis of self-renewal of WT and *Tet2*$^{-/-}$ HSPCs before and after light stimulation.

(a) The gating strategy for LOCa3-expressing HSPCs marked by Lin^{-} c-Kit^{+} Sca-1^{+}. In brief, after the exclusion of cell debris using side-scatter (SSC) and forward-scatter (FSC), mCherry-positive cells were gated, then lineage negative cells were gated to identify the c-Kit^{+} Sca-1^{+} double positive population.

(b) Quantification of the LSK population of HSPCs by flow cytometry. Normal (WT) or *Tet2*-KO HSPCs were analyzed before and after light stimulation. HSPCs were subjected to *in vitro* expansion for 5 days. The self-renewal ability was gauged by the frequency of the LSK cell population marked by Lin^{-} c-Kit^{+} Sca-1^{+} (red box).

Supplementary Table 1. Comparison of LOCa3 with other optogenetic tools used for interrogating Ca^{2+} signaling.

Names	Photosensitive module	Engineering templates	$t_{1/2;}$ ON (sec)	$t_{1/2;}$ OFF (sec)	Ca^{2+} selectivity	Notes
ChR2[1]	opsin	Microbial opsin	~0.002	~0.01	NO	Permeable to H^+, Na^+, K^+, Ca^{2+}, Mg^{2+}; fast kinetics (ms); widely used in neuroscience and excitable tissues
LOCa3	LOV2	ORAI1	~34-49	~57	YES	Not endogenous STIM- or ORAI-level dependent; low basal activity; high photosensitivity
Opto-CARC variants[2, 3]	LOV2	STIM1	~10-23	~23-35	YES	Depending on endogenous ORAI levels
	CRY2 + CIBN	STIM1	23.4± 2.6	153.0± 26.2	YES	Depending on endogenous ORAI levels
	iLID (LOV2-ssrA + sspB)	STIM	28.5± 3.2	48.6± 5.4	YES	Depending on endogenous ORAI levels
BACCS[4]	LOV2	STIM1 and/or *Drosophila* ORAI	<30	30-60	YES	Endogenous ORAI level dependent (human version) or independent (*Drosophila* version)
OptoSTIM1[5]	CRY2	STIM1	~43-65	~274-383	YES	Endogenous ORAI level dependent, relatively slower deactivation speed than LOV2-based tools
monSTIM1[6]	CRY2 (E281A)	STIM1	24.8 ± 1.0	513.5 ± 25.8	YES	Endogenous ORAI level dependent; an improved version of OptoSTIM1 with less basal activity and improved photosensitivity

Supplementary Table 2. Primers used in this study.

Name	Sequence
mKate2-P2A-NheI-for	CGG GCTAGC ATGGTGAGCGAGCTGATTAA
mKate2-P2A-BamHI-rev	CGG GGATCC GAGCTCGGTACCGGGGCCGGGGTTCTCCTC
Orai1-BglII-for	CGG AGATCT GGAAGCGGTATGCATCCGGAGCCCGCCCCG
Orai1-XhoI-rev	CGG CTCGAG CTA GGCATAGTGGCTGCCGGGCGT
Orai1-P245T	TCGACCACCATCATGGTTACCTTCGGACTGATCTTTATC
Orai1-H134A	ACA GTGCTGGTGGCTGTG GCCCTGTTTGCGCTC ATGATC
Orai1-L261A/V262N	CACTTCTAC CGCTCAGCGAATAGC CATAAGACTGACCGA
Orai1-L261A/V262N/H264G/K265A	GTCCACTTCTACCGCTCAGCGAATAGCGGCGCGACTGACCGACAGTTCCAGGAG
LOV2-Hifi-for	AAGGAGTCAGGATCCGGGTTGGCTACTACACTTGAACGT
LOV2-hifi-rev	CTCATGGGGTCCGGACCCAAGTTCTTTTGCCGCCTCATC
Orai-V-hifi-for	GGGTCCGGACCCCATGAGCGCATGCACCG
Orai-V-hifi-rev	CCCGGATCCTGACTCCTTGACCGAGTTGAGATTGTG
Orai1-65-BglII-for	CGG AGATCT ATGAGCCTCAACGAGCACTCC
Orai1-286-XbaI-rev	CGG TCTAGA CTA CAGCTGGTCCTGTAAGCGGGC
Orai1-XhoI-for	CGG CTCGAG ATGCATCCGGAGCCCGCCCCG
Orai1-EcoRI-rev	CCG GAATTC C GGCATAGTGGCTGCCGG
Orai1-EcoRI-for	CGG GAATTC GCCACC ATGCATCCGGAGCCCGCCCCG
Orai1-NotI-rev	CGG GCGGCCGC CTA GGCATAGTGGCTGCCGGGCGT
pGL-hifi-for	TTACCACCATAGCCCGGGGGCCGCGACTCTAGAGTCGGG
pGL-hifi-rev	ATTTTCCATCTCGAGGGTGGCTTTACCAACAGTAC
MLKL-hifi-for	ACCCTCGAGATGGAAAATTTGAAGCATATT
MLKL-hifi-rev	CCCGGGCTATGGTGGTAAATACTGCCTCAA
Orai-pValium20-XbaI-for	CGG TCTAGA ACCATGGGTCATCCGGAGCCCGCCCCG
Orai-pValium20-EcoRI-rev	CGG GAATTC CTAGGCATAGTGGCTGCCGGGCGT
Orai1-Flag-GSGS-for	GATGACGACAAG GGCAGCGGCAGC GGCCAG CCAAGGCCCACCAG
Orai1-Flag-GSGS-rev	GTCTTTGTAGTC GCTGCCGCTGCC TGGCTGCTTCTTGAGGGGCAAGAACTT

Supplementary References

1. Nagel, G. et al. Channelrhodopsin-2, a directly light-gated cation-selective membrane channel. *Proc Natl Acad Sci U S A* **100**, 13940-13945 (2003).
2. He, L. et al. Near-infrared photoactivatable control of Ca(2+) signaling and optogenetic immunomodulation. *Elife* **4** (2015).

3. Ma, G. et al. Optogenetic engineering to probe the molecular choreography of STIM1-mediated cell signaling. *Nat Commun* **11**, 1039 (2020).
4. Ishii, T. et al. Light generation of intracellular Ca(2+) signals by a genetically encoded protein BACCS. *Nat Commun* **6**, 8021 (2015).
5. Kyung, T. et al. Optogenetic control of endogenous Ca(2+) channels in vivo. *Nat Biotechnol* **33**, 1092-1096 (2015).
6. Kim, S. et al. Non-invasive optical control of endogenous Ca(2+) channels in awake mice. *Nat Commun* **11**, 210 (2020).
