# Supplementary material for: Engineering of a bona fide light-operated calcium channel
Source: Nat Commun. 2021 Jan 11;12:164. doi: 10.1038/s41467-020-20425-4 (PMC7801460; doi:10.1038/s41467-020-20425-4)
Supplement: Supplementary file 3 — Description of Additional Supplementary Files [file 41467_2020_20425_MOESM3_ESM.pdf]

Title: Supplementary Movie 1
Description: Spatial control of calcium influx by LOCa3 in mammalian cells. Time-lapse imaging of two HeLa cells co-expressing LOCa3 and the red colored calcium indicator jRCaMP1b. Sequential localized photostimulation (488 nm laser; 0.5 % output) was applied to the cells.

Title: Supplementary Movie 2
Description: Wild type flies show fast climbing ability in the climbing assay. Ten flies (31-day-old males) were provoked by tapping the bottom of the glass cylinder housing them and their climbing ability was monitored by counting the number of flies crossing the 10-cm line within 10 sec. About six flies crossed the marked line for each group, with no significant difference noted between the dark and lit groups. The blue light treatment was given as 10 min for each hour per day until assayed (pulsed LED at 470 nm with a density of 40 µW/mm2).

Title: Supplementary Movie 3
Description: A β42-expressing flies showed a severe climbing defect. None of the Aβ42 flies could cross the 10-cm line in 10 sec. Flies were the same age and received the same photostimulation protocol as described in Movie 2.

Title: Supplementary Movie 4
Description: Light-dependent rescue of the climbing ability of A β42 transgenic flies with LOCa3 co-expression. 31-day-old male Aβ42 flies expressing LOCa3 when treated with blue light showed a significant improvement in the climbing ability compared to flies reared in the dark. Six LOCa3 flies in the lit group traveled across the 10-cm line, whereas only two flies in the dark group made it within 10 sec
