# Supplementary material for: Engineering of a bona fide light-operated calcium channel
Source: Nat Commun. 2021 Jan 11;12:164. doi: 10.1038/s41467-020-20425-4 (PMC7801460; doi:10.1038/s41467-020-20425-4)
Supplement: Supplementary file 8 — Reporting Summary [file 41467_2020_20425_MOESM8_ESM.pdf]

nature research

Corresponding author(s): Yubin Zhou

Last updated by author(s): Nov 19, 2020

Reporting Summary

Nature Research wishes to improve the reproducibility of the work that we publish. This form provides structure for consistency and transparency in reporting. For further information on Nature Research policies, see Authors & Referees and the Editorial Policy Checklist .

Statistics

For all statistical analyses, confirm that the following items are present in the figure legend, table legend, main text, or Methods section.

n/a	Confirmed	
☐	☒	The exact sample size (*n*) for each experimental group/condition, given as a discrete number and unit of measurement
☐	☒	A statement on whether measurements were taken from distinct samples or whether the same sample was measured repeatedly
☐	☒	The statistical test(s) used AND whether they are one- or two-sided<br>*Only common tests should be described solely by name; describe more complex techniques in the Methods section.*
☒	☐	A description of all covariates tested
☒	☐	A description of any assumptions or corrections, such as tests of normality and adjustment for multiple comparisons
☐	☒	A full description of the statistical parameters including central tendency (e.g. means) or other basic estimates (e.g. regression coefficient) AND variation (e.g. standard deviation) or associated estimates of uncertainty (e.g. confidence intervals)
☒	☐	For null hypothesis testing, the test statistic (e.g. *F*, *t*, *r*) with confidence intervals, effect sizes, degrees of freedom and *P* value noted<br>*Give P values as exact values whenever suitable.*
☒	☐	For Bayesian analysis, information on the choice of priors and Markov chain Monte Carlo settings
☒	☐	For hierarchical and complex designs, identification of the appropriate level for tests and full reporting of outcomes
☒	☐	Estimates of effect sizes (e.g. Cohen's *d*, Pearson's *r*), indicating how they were calculated

Our web collection on statistics for biologists contains articles on many of the points above.

Software and code

Policy information about availability of computer code

Data collection	The fluorescence imaging data were obtained by using Nikon NIS-Elements AR (Nikon, version 4.51.00) and high-throughput imaging data were acquired by IN Cell Analyzer 6000 (GE-Healthcare Life Sciences); the FACS data were obtained using the LSRII cytometer (BD Biosciences. Inc); Luciferase data were collected using the Cytation 5 Cell Imaging Multi-Mode Reader and Gen5 software (BioTek); HEKA EPC 10 USB double patch amplifier was used for electrophysiological measurements. Calcium imaging in Drosophila brain was acquired by using an LSM 880 NLO confocal microscopy (Zeiss).Single-cell intracellular Ca2+ imaging were controlled by SlideBook6.0 software (Intelligent Imaging Innovations, Inc.). Whole-cell Pathclamping data were collected with Pathmaster software (HEKA Instruments Inc.)
Data analysis	Imaging data analyses were performed using the Nikon NIS-Elements AR Analysis (version 4.51.00). FlowJo (v10.5.3) was used for all flow cytometry analysis. High-throughput imaging data were analyzed by using Pipeline Pilot (9.5). Data analysis for Ca2+ imaging in the Drosophila brain were carried out with Matlab R2014a software (MathWorks). GraphPad Prism8 (v8.3.0) was used to generate all graphs and to perform statistical analyses. Statistical analysis was performed using two tailed unpaired Student's t-test.

For manuscripts utilizing custom algorithms or software that are central to the research but not yet described in published literature, software must be made available to editors/reviewers. We strongly encourage code deposition in a community repository (e.g. GitHub). See the Nature Research guidelines for submitting code & software for further information.

Data

Policy information about availability of data

All manuscripts must include a data availability statement. This statement should provide the following information, where applicable:

- Accession codes, unique identifiers, or web links for publicly available datasets
- A list of figures that have associated raw data
- A description of any restrictions on data availability

Supplementary Data are available online. The source data underlying Figs 1b, d, e, h, 2a, c, d, e, f, 3c, e, f, h, j, 4b, c, d and Supplementary Figs. 1a, b, c, 2a, c, 3a, b, c, 4a, b, c, d and 5a, b, c are provided as a Source Data file. The plasmids and all other data are available from the corresponding author upon reasonable request. Source data are provided with this paper.

Field-specific reporting

Please select the one below that is the best fit for your research. If you are not sure, read the appropriate sections before making your selection.

[x] Life sciences [ ] Behavioural & social sciences [ ] Ecological, evolutionary & environmental sciences

For a reference copy of the document with all sections, see nature.com/documents/nr-reporting-summary-flat.pdf

Life sciences study design

All studies must disclose on these points even when the disclosure is negative.

Sample size	No statistical tests were performed to predetermine the sample sizes. Sample sizes were chosen on the basis of an initial pilot experiment and further based on similar experiments reported in previous publications, "Ma, G., He, L., Liu, S. et al. Optogenetic engineering to probe the molecular choreography of STIM1-mediated cell signaling. Nat Commun 11, 1039 (2020)". At least 3 biological replicates were performed for most cases, unless otherwise noted in the figure legends.
Data exclusions	No data were excluded from the analysis.
Replication	All the experiments were successfully replicated three times. The number of replicates performed is indicated in each figure legend, where applicable.
Randomization	All the samples and controls were treated side-by-side using the identical protocols and the samples in the same experiment group were random. The detected cells or imaging views were randomly selected.
Blinding	The investigators were blinded to group allocation during data collection and analysis.

Reporting for specific materials, systems and methods

We require information from authors about some types of materials, experimental systems and methods used in many studies. Here, indicate whether each material, system or method listed is relevant to your study. If you are not sure if a list item applies to your research, read the appropriate section before selecting a response.

Materials & experimental systems

n/a	Involved in the study	
[ ]	[x]	Antibodies
[ ]	[x]	Eukaryotic cell lines
[x]	[ ]	Palaeontology
[x]	[ ]	Animals and other organisms
[x]	[ ]	Human research participants
[x]	[ ]	Clinical data

Methods

n/a	Involved in the study	
[x]	[ ]	ChIP-seq
[ ]	[x]	Flow cytometry
[x]	[ ]	MRI-based neuroimaging

Antibodies

Antibodies used

Biotin anti-mouse Lineage Panel (Ter-119, Gr1, Mac-1, B220, CD3) #133307, Biolegend, Dilution 1:50
Streptavidin-eFluor®450, #48-4317-82, ThermoFisher Scientific, Dilution 1:400
CD117 (c-Kit) Monoclonal Antibody (2B8), APC, #17-1171-82, ThermoFisher Scientific, Dilution 1:200
Ly-6A/E (Sca-1) Monoclonal Antibody (D7), FITC, #11-5981-82, ThermoFisher Scientific, Dilution 1:200
Anti-mouse Alexa Fluor-488 IgG, # A-11029, ThermoFisher Scientific, Dilution 1:1000
FLAG monoclonal antibody, # F3165, Sigma-Aldrich, Dilution 1:300

Validation

All antibodies were commercially available and were validated by manufacturers, in previous publications, and in this study.

Biotin anti-mouse Lineage Panel:
validation can be found in the manufacturer's website https://www.biolegend.com/en-us/products/biotin-anti-mouse-lineage-panel-7611?GroupID=GROUP20
Used in several studies: Morimoto Y, et al. 2018. Immunity. 49:134; rizotte–Lake M, et al. 2018. Immunity. 49:1103; Guo L, et al. 2020. Nat Commun.0.972222222.

Streptavidin-eFluor®450:
https://www.thermofisher.com/order/catalog/product/48-4317-82#/48-4317-82
Fang, J et al. Nat Immunol 18, 236–245 (2017).

CD117 (c-Kit):
https://www.thermofisher.com/antibody/product/CD117-c-Kit-Antibody-clone-2B8-Monoclonal/17-1171-82
Used by: Chaudhury S,O'Connor C, et al. Nat Commun. 2018 Dec 11;9(1):5280. Somervaille TC,et al. Cell Stem Cell. 2009 Feb 6;4 (2):129-40.

Ly-6A/E (Sca-1) Monoclonal Antibody (D7), FITC:
https://www.thermofisher.com/antibody/product/Ly-6A-E-Sca-1-Antibody-clone-D7-Monoclonal/11-5981-82
Wang J,et al. Nat Commun. 2019 Oct 31;10(1):496; Moretti FA, et al. Elife. 2018 Sep 6;7:e35816.

Anti-mouse Alexa Fluor-488 IgG
https://www.thermofisher.com/antibody/product/Goat-anti-Mouse-IgG-H-L-Highly-Cross-Adsorbed-Secondary-Antibody-Polyclonal/A-11029
Gilsbach R, Nat Commun. 2018 Jan 26;9(1):391; Xiong G et al. Elife. 2017 Mar 23;6:e22871.

FLAG monoclonal antibody
https://www.sigmaaldrich.com/catalog/product/sigma/f3165?lang=en&region=US
Annie M Sriramachandran et. al, Nature communications, 10(1), 3678 (2019-8-17); Jose A Martina et. al. Autophagy, 8(6), 903-914 (2012-5-12).

Eukaryotic cell lines

Policy information about cell lines

Cell line source(s)	HeLa, HEK293T, MCF7, A549, U87, HSkMC and SH-SY5Y were obtained from ATCC. HeLa cells stably expressing NFAT(1-460)-GFP was generated by our lab.
Authentication	Authenticated by the vendor with STR profiling.
Mycoplasma contamination	Mycoplasma contamination was tested by using the LookOut PCR detection kit from SigmaAldrich. All cell lines used were mycoplasma free.
Commonly misidentified lines (See ICLAC register)	No commonly misidentified cell lines were used.

Flow Cytometry

Plots

Confirm that:

- [x] The axis labels state the marker and fluorochrome used (e.g. CD4-FITC).
- [x] The axis scales are clearly visible. Include numbers along axes only for bottom left plot of group (a 'group' is an analysis of identical markers).
- [x] All plots are contour plots with outliers or pseudocolor plots.
- [x] A numerical value for number of cells or percentage (with statistics) is provided.

Methodology

Sample preparation

Retrovirus was packaged in plat-E cells by co-transfection of the MSCV-IRES-mCherry based vector and the PCL-ECO helper plasmid 49. 4X107 bone marrow cells were harvested from the femurs of wild-type or Tet2-/- C57BL/6 mice. After red cell lysis, the bone marrow cells were stained with a panel of biotin-conjugated antibodies (1:10 dilution). Specifically, antibodies against Ter-119 (BioLegend Ter119, 1:50), Gr1 (BioLegend RB6-8C5, 1:50), Mac-1 (BioLegend M1/70, 1:50), B220(BioLegend RA3-6B2, 1:50), CD3 (BioLegend 17A2, 1:50) surface antigen were mixed as a cocktail for lineage labeling. The cells were washed once and then incubated with anti-biotin microBeads (cat number: 130-090-485) by following the manufacturer's instructions, followed by negative selection using the MACS LS Columns (cat number: 130-042-401). The flow-through was considered as the Lineage negative fraction, also known as HSPCs. The Lin- cells was cultured in StemSpan SFEM (StemCell Technologies) supplemented

with 10% FBS and recombinant murine IL-3 (10 ng/ml) (PeproTech, Catalog Number: 213-13), recombinant murine IL-6 (10 ng/ml) (PeproTech Catalog Number:216-16), recombinant murine IL-6SCF (50 ng/ml) (PeproTech, Catalog Number:250-03). Twenty-four hours later, WT and Tet2-/- HSPC cells were infected with a retrovirus expressing LOCa3. Cells were infected twice with 24 hr in between to boost the transduction efficiency.

For ex vivo culture, LOCa3-expressing cells were plated at 2x105/ml and grown for 3-5 days in StemSpan SFEM (StemCell Technologies) supplemented with recombinant murine IL-3 (10 ng/ml) (PeproTech, Catalog Number: 213-13), recombinant murine IL-6 (PeproTech Catalog Number:216-16), and recombinant murine SCF (50 ng/ml) (PeproTech, Catalog Number:250-03). Cells were kept in the dark or stimulated with blue light for 30 min every day.

For the bone marrow hematopoietic progenitor stem cell (HSPCs) marker by LSK (Lin-c-Kit+Sca-1+) analysis, a panel of biotin-conjugated antibodies of cocktail for lineage labeling followed by secondary streptavidin-eFluor®450 (ThermoFisher Scientific 48-4317-82, 1:400) staining, c-Kit APC (ThermoFisher Scientific 2B8, 1:200), Sca-1 FITC (ThermoFisher Scientific D7, 1:200)

Instrument	All the data were collected on a LSRII flow cytometer (BD Biosciences).
Software	BD FACSDiva and FlowJo v10.5.3
Cell population abundance	Lin- population abundance is about 5% of the live cell population.<br>LSK population abundance ranged between 14% and 45% of the Lin- population.
Gating strategy	An example of the gating strategy used is shown in Supplementary Fig. 2a

☒ Tick this box to confirm that a figure exemplifying the gating strategy is provided in the Supplementary Information.
